# The concept of motivation and its implication in additive disorders

**DOI:** 10.1192/j.eurpsy.2021.1525

**Published:** 2021-08-13

**Authors:** A.B. Medeiros, J. Teixeira

**Affiliations:** 1 Serviço De Psiquiatria E Saúde Mental, Hospital Garcia de Orta, E.P.E., Almada, Portugal; 2 Clínica 4 - Unidade De Alcoologia E Novas Dependências, Centro Hospitalar Psiquiátrico de Lisboa, Lisboa, Portugal

**Keywords:** motivation, Addictive disorders, Motivational Interview, Dopamine

## Abstract

**Introduction:**

The word motivation derives from the Latin movere, which means to move. In psychiatry, it is an isolated phenomenon found in the substrate of several pathologies, and may be part of an heterogeneous dimensional spectrum. However, there is no unique definition for it, nor a targeted approach. In addictive disorders motivation gains a fundamental role, both as a precipitant of abuse as in its withdrawal.

**Objectives:**

To review the literature about the concept of motivation and its implications on the psychopathology, especially on addictive disorders.

**Methods:**

Narrative review on PubMed/MEDLINE, using the keywords “motivation” AND “psychopatology” AND “addiction”. Articles in English and Portuguese were included.

**Results:**

Three main perspectives were found addressing the concept of motivation in psychopathological terms: psychological, neurobiological and phenomenological. The first describes motivation as the energizing of behaviour in pursuit of a goal. Neurobiology says motivational drive is dependent on the concentration of extrasynaptic dopamine. In phenomenological terms, the concept stands for the web of solicitations that make a certain situation feel in a certain way for the subject. In addictive disorders, learning about what leads to reward, exaggeration in representing those values, and dominance in being guided by those representations lead to alterations on motivation mechanisms.

**Conclusions:**

Motivation is described from different perspectives. Although it is recognized as a fundamental piece in addictive disorders, besides motivational interview model, there are no pharmacological approaches aimed to improving motivation. The recognition of motivation as a concrete psychopathological alteration, and its measure through psychopathological instruments, could optimize the patient’s approach.

